# Effect of surgical shoes on brake response time after first metatarsal osteotomy—a prospective cohort study

**DOI:** 10.1186/s13018-016-0350-9

**Published:** 2016-01-20

**Authors:** Dietmar Dammerer, Matthias Braito, Rainer Biedermann, Michael Ban, Johannes Giesinger, Christian Haid, Michael C. Liebensteiner, Gerhard Kaufmann

**Affiliations:** Department of Orthopaedics, Medical University of Innsbruck, Anichstr. 35, A-6020 Innsbruck, Austria; Department of Psychiatry, Medical University of Innsbruck, Anichstr. 35, A-6020 Innsbruck, Austria

**Keywords:** Hallux valgus, Surgical shoe, Foot orthoses, Driving safety, Brake response time

## Abstract

**Background:**

The aim of this study is to assess patients’ driving ability when wearing surgical shoes following right-sided first metatarsal osteotomy.

**Methods:**

From August 2013 to August 2015, 42 consecutive patients (mean age 54.5 years) with right-sided hallux valgus deformity underwent first metatarsal osteotomy. Patients were tested for brake response time (BRT) 1 day preoperatively (control run) and at 2 and 6 weeks postoperatively. Two different types of foot orthosis were investigated. BRT was assessed using a custom-made driving simulator.

**Results:**

Preoperative BRT was 712 msec (standard deviation (SD), 221 msec). BRT was significantly slower at all tested postoperative times than preoperatively (*p* < 0.001). The patients showed significant impaired brake response time when wearing surgical shoes. Mean global American Orthopaedic Foot and Ankle Society (AOFAS) outcome score and AOFAS pain and alignment subscores increased postoperatively (*p* < 0.001).

**Conclusions:**

From our findings, we recommend driving abstinence for a minimum of 6 weeks postoperatively when using a surgical shoe after bunionectomy. However, patients should have sufficient recovery, exercise, and training before resuming driving a car, because safety is always a priority.

**Trial registration:**

ClinicalTrials.gov, NCT02354066

## Background

Painful hallux valgus deformity is a frequent clinical problem and a disabling condition that may require surgical corrections [1, 2]. Surgeons are often confronted with the question whether and when to allow their patients to resume driving postoperatively.

When patients are temporarily unable to drive a car, they lose their independence and quality of life, which may have serious social and economic consequences for them. The advice given to such patients to resume driving lacks supportive evidence as few scientific data are available on this specific subject [3]. In addition, of the various factors that constitute driving performance, brake response time (BRT) is considered one of the most important factors responsible for driving safety [4]. To the best of our knowledge, there is to date no evidence on the effect that right-sided foot orthoses have on BRT following first metatarsal osteotomy. Numerous studies have investigated driving ability in the context of orthopedic surgical procedures [5–8].

Egol et al. investigated driving ability following surgical treatment of ankle fractures and showed that brake response times did not recover to control values until 9 weeks after surgery [9]. Only Holt et al. studied the influence on BRT after first metatarsal osteotomy and concluded that 6 weeks after surgery BRT is similar to that of healthy individuals [3]. First metatarsal osteotomy is considered a minor procedure compared to other orthopedic procedures (e.g., arthroplasty), but the right foot is potentially more disabling when it comes to applying substantial amounts of pressure to the forefoot during braking [3]. However, none of the mentioned studies investigated the influence of various surgical shoes.

Given the lack of evidence in the current literature, we conducted a prospective cohort study to assess the effect of various right-sided foot orthoses on the driving ability of patients undergoing a first metatarsal osteotomy with a symptomatic right-sided hallux valgus deformity. We hypothesized that BRT would show statistically significant differences between the two types of foot orthosis and the preoperative baseline values.

## Methods

### Materials

The local ethics committee approved the study protocol, and written informed consent was obtained from all patients before participation. The study is registered in a public trials registry. The national authorities approved all described devices.

#### Treatment

From August 2013 to August 2015, 42 consecutive patients (34 women, 8 men) were recruited for this prospective cohort study. All patients had a symptomatic hallux valgus deformity of the right foot with no fixed deformities of the lesser toes. To be included in the study, patients had to have a valid driver’s license, had to use the right foot exclusively for accelerating and braking, and must have been free of any medical condition that could impair their ability to drive. Patients who were taking medication that could affect reaction time or had a history of alcohol or drug abuse, a central nervous system disorder such as epilepsy, a psychiatric disorder, or musculoskeletal disease, as well as participants with any recent (previous 3 months) surgery of the right lower limb or any visual acuity disorder were excluded.

In all cases, a chevron or scarf osteotomy of the first metatarsal and a lateral release of the first metatarsophalangeal joint (with or without an Akin osteotomy of the greater toe) were performed depending on the severity of the hallux valgus deformity and the preoperative intermetatarsal and hallux valgus angle. All osteotomies were stabilized with one or two screws or intraosseous seams. Postoperative treatment was standardized. Simple wound dressings were applied, and all patients were advised to wear a surgical shoe with full weight bearing as tolerated for 6 weeks.

#### Tests

For brake response time measurements, all patients were given the same standardized instructions following the study protocol and each patient was asked to evaluate his own driving frequency (years of driving experience and distance traveled in kilometers per year).

BRT measurements were performed with a validated experimental apparatus according to the protocols of previous studies performed by our group and other researchers in the field [5, 6, 8, 10–12].

**Fig. 1:**
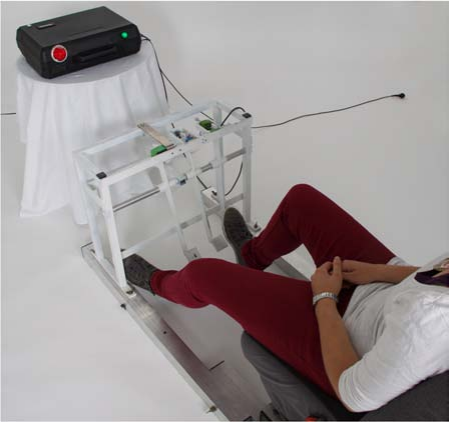
Custom-made apparatus for measuring brake response time (BRT). Pedals from left to right: coupler [clutch] pedal; brake pedal; accelerator pedal. External box: red light (visual stimulus initiating the braking procedure); green light (indicating that the accelerator is fully depressed—subject not in a ‘ready-to-brake fashion’)

An adjustable car seat fixed on a frame with three hanging pedals (accelerator, coupler [clutch], and brake pedal) mounted on rubber-damped pivots (Fig. 1) was used. The position of the seat was adjustable with regard to seat inclination, headrest, and seat-pedal distance to simulate the patient’s customary driving position [11, 13, 14]. At a constant distance in front of the patient, an external box containing the electric logic circuit, a red light and a green signal light, was positioned. A measurement sequence started when the patient depressed the accelerator pedal fully and the green light lit up. After an interval of 5 to 30 s (investigator’s discretion), the investigator pushed an external trigger concealed from the patient’s view that activated the red signal light and the electronic clock. The participants had been instructed to depress the brake pedal as quickly as possible with the right foot when the red signal light lit up. The time interval (measured in milliseconds; msec) between the time when the red signal was switched on and the time when the brake pedal was depressed was registered and denoted the BRT. The abovementioned interval between tests was deemed to not be influenced by a fatigue effect in a previous study [11].

All tests were performed exclusively with the right foot. Each participant performed three practice trials to familiarize him- or herself with the foot orthoses before starting the experiment. BRT was measured 20 times for each foot orthosis model.

**Fig. 2:**
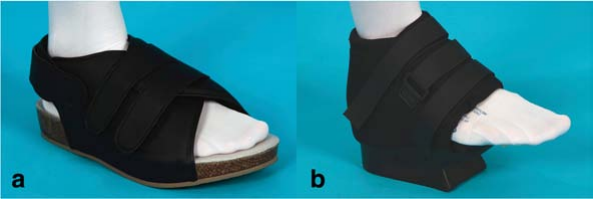
Investigated foot orthoses. A hallux valgus shoe (**a**; with rounded and stiffened sole) and a typical forefoot relief shoe (**b**) were used postoperatively

We measured BRT at three different times: the day before the operation without a foot orthosis (control run; patients were asked to wear footwear of their own choice) (1), at 2 weeks (2), and 6 weeks (3) after the osteotomy while wearing a typical postoperative hallux valgus shoe (HVS; Hallux Valgus Shoe, Ofa GmbH, Bamberg, Germany) as well as a forefoot relief shoe (FRS; GloboPed®, Bauerfeind AG, Zeulenroda-Triebes, Germany). BRT was also tested without foot orthosis, namely with the patients wearing their own footwear and 6 weeks postoperatively. Fixation of the foot orthoses was standardized and performed by the same investigator. The surgical shoes are shown in Fig. 2.

Preoperatively and at the 6-week follow-up, the patients underwent a standardized physical examination with X-ray. The patients were allowed to wear footwear of their choice when the osteotomy was deemed to be healed from its radiographic appearance. In addition, an assessment using the AOFAS (American Orthopaedic Foot and Ankle Society’s) Hallux Metatarsalphalangeal-Interphalangeal Scale was performed preoperatively and 8 weeks postoperatively.

#### Analysis

Sample characteristics are given as mean, standard deviation, range, and frequency. For BRT, we calculated means, standard deviations, and 95 % confidence intervals separately for each time and condition. In addition, we provided percentages of measurements below 700 msec, between 700 and 1500 msec, and above 1500 msec. The 700 and 1500-msec thresholds were chosen because several road authorities recommend absolute maximum BRT values between 700 and 1500 msec [15–17]. Since BRT showed a skewed distribution, we log transformed the variable for conducting statistical tests requiring a normal distribution. We used a linear mixed model to investigate the association between BRT and time, condition, and patient characteristics. Pair-wise comparisons with Bonferroni correction were performed to compare baseline BRT measurements and BRT at subsequent times. Comparisons of pre- and postop AOFAS scores were based on a *T* test for dependent samples.

## Results

Patients’ sociodemographic characteristics and driving experience are presented in Table 1. Forty-two patients (34 women, 8 men) with a mean age of 54.5 years (standard deviation (SD), 12.3 years; range, 20–78 years) were included in this study. All patients completed the study. Two patients were not able to undergo the 2-week follow-up BRT measurement because of pain.

**Table 1:** Sociodemographic characteristics and driving experience (*N* = 42; 34 women, 8 men)

	Mean	SD	Range
Age	54.5	12.3	20–78
Years of driving experience	31.0	11.2	2–50
Distance traveled (km/year)	10,568	9307	500–36,000

*SD* standard deviation

We performed 22 chevron, 11 scarf, 5 combined chevron/Akin, and 4 combined scarf/Akin osteotomies. The type of osteotomy performed did not have a significant effect on brake response time.

Preoperative BRT was 712 msec (SD, 221 msec). A statistically significant difference between the preoperative control run and all postoperative BRT measurements was found. Pre- and postoperative BRT measurements are shown in Table 2. The proportion of patients above or below the threshold of 700-msec BRT is shown in Table 3.

**Table 2:** Brake reaction time measured in millisecond with the various orthoses—compared to the control run

Type of knee brace	Mean	SD	95 % CI	*p* values, comparison with control run
Preop no orthosis (control run)	712	221	697–727	–
HVS postop (2 weeks)	804	275	784–825	*p* < 0.001
FRS postop (2 weeks)	841	245	823–860	*p* < 0.001
HVS postop (6 weeks)	750	200	736–765	*p* = 0.003
FRS postop (6 weeks)	811	221	795–827	*p* < 0.001
PostOP no orthosis (6 weeks)	769	213	752–785	*p* < 0.001

SD standard deviation)

**Table 3:** Proportion of patients above or below the threshold of 700 msec BRT

Percent (%)		
	<700 msec	>700
Preop (control run)	56.3	43.7
HVS postop (2 weeks)	43.7	56.3
FRS postop (2 weeks)	31.4	68.6
HVS postop (6 weeks)	50.9	49.1
FRS postop (6 weeks)	39.5	60.5
Postop no orthosis (6 weeks)	47.0	53.0

Mean brake response time was shorter for the men (689 msec, *p* < 0.001) than for the women (853 msec). The men had significantly more driving experience than did the women (*p* < 0.001), and regular drivers recorded significantly better brake response times (*p* < 0.001). We did not find a significant correlation between median BRT and age (*r* = 0.048; *p* = 0.768).

Mean global AOFAS outcome score and AOFAS pain and alignment subscores increased postoperatively (*p* < 0.001; Table 4).

**Table 4:** Results of AFOAS 1st Forefoot Ray Scale. Significantly better postoperative outcome in the subscores for pain, alignment, and global. *p* values of assessment preoperative und 8 weeks postoperative

	Pain	Function	Alignment	Global
AOFAS preop	23.6 (±7.9)	31.0 (±9.1)	5.1 (±4.2)	58.6 (±15.0)
AOFAS postop (8 weeks)	31.4 (±8.7)	31.5 (±5.9)	13.4 (±3.0)	76.3 (±13.7)
*p* values	<0.001	0.771	<0.001	<0.001

By the 8-week follow-up examination, no patient had a clinically important postoperative complication and all patients had returned to wearing their own footwear.

## Discussion

Our results reveal a statistically significant impairment in BRT at all postoperative measurement times when wearing the investigated foot orthoses. Mean difference in BRT between the measurements 2 weeks postoperatively with the HVS and FRS as compared to the preoperative control run without foot orthosis showed a mean increase in BRT of 92 msec (HVS) and 129 msec (FRS). This translates to an additional braking distance of approximately 10 ft. (3.0 m) or 14 ft (4.3 m), respectively, at a driving speed of 75 mph (120 km/h). The measurements made 6 weeks postoperatively without a foot orthosis also showed a statistically significant increase in BRT, but this small increase in the mean brake response time, seen 6 weeks after surgery, is not large enough to advocate advising patients who have undergone corrective surgery for a hallux valgus deformity to refrain from driving beyond 6 weeks after surgery.

Although we found statistically significant impairment at all postoperative test occasions, the relevance seems to be minor with regard to the broad range of thresholds published by road authorities. A number of road authorities’ recommended different values for maximum BRT, ranging from 700 to 1500 msec [15–17]. To the best of our knowledge, no single value has been generally accepted with regard to declaring a driver safe. Banning patients from driving would have a great impact on social life and the daily to-do’s. Therefore, we believe that no driving abstinence must be recommended for patients after first metatarsal osteotomy beyond 6 weeks after surgery (BRT impairment is statistically significant but not clinically relevant).

Brake response times and recommendations for driving resumption have been investigated in the context of various orthopedic procedures, but only a few experimental studies have looked at BRT during partial orthopedic immobilization of the right lower limb [11, 18, 19]. Giddins and Hammerton investigated legal and economic concerns by reviewing laws and driver license requirements in the UK and surveying major automobile insurance companies [20]. They found that drivers in the UK were covered by their insurance if the driver resumed driving with the permission of a doctor [20]. Therefore, Giddins and Hammerton stated that doctors should avoid giving detailed legal advice to patients [20]. In the USA, the National Highway Traffic Safety Administration recommends not driving with any splint or immobilization device, but this is a guideline and not a law [21]. However, studies have shown that patients often continue to drive despite immobilization of an extremity, thus demonstrating the importance of mobility for patients [21].

Holt et al. [3] investigated the effect on BRT following first metatarsal osteotomy and suggested that patients may safely resume driving 6 weeks postoperatively [3]. In their study, patients did not wear a surgical shoe and the authors excluded the highest and the lowest measured values. Furthermore, it should be mentioned that the study by Holt et al. involved only 28 patients, whereas our study comprised 42 patients and all measured BRT values. Performing an emergency braking maneuver involves exerting a substantial amount of force with the forefoot in order to rapidly depress the brake pedal [3]. We agree with Holt et al., who stated that waiting 6 weeks is sensible in that it allows a sufficient period of time for the osteotomy to heal and sustain that type of force without displacement [3]. In our study, the patients showed a slight increase in BRT at the 6-week measurement without foot orthosis as compared with the 6-week BRT results when wearing the HVS or FRS. This might be explained by patient’s potential concerns about putting full weight on the right foot in a patient-chosen shoe for the first time after the period when only partial weight was put on the foot in a surgical shoe. Nevertheless, it should be emphasized that the time when a patient resumes driving is ultimately the patient’s responsibility, even if physicians offer recommendations following orthopedic surgery.

The ability to drive a car safely is multifactorial, and BRT is only one of the several important factors that should be considered [3]. Surgeons should take care when giving advice regarding driving a car following foot surgery. It might be speculated whether there is a specific amount of BRT increase that would result in a greater accident likelihood. However, to our knowledge, there is no evidence to quantify the relation between BRT increase and accident risk. We performed a PubMed literature search and found no studies regarding the relationship between impaired BRT, on the one hand, and accident risk, on the other hand [11]. We therefore recommend that additional studies be performed on this specific issue.

Brake response time, as defined in our study, covers (1) neurologic reaction time, (2) foot transfer time, and (3) time necessary to exert pressure on the pedal. This is well in line with previous research in the field of driving safety [5, 11]. Reaction is considered a complex task [22–24]. It includes various psychomotor processes and is defined as the amount of time an individual takes to respond and complete a movement after a stimulus has been presented [22–24]. In our study, brake reaction time was defined as the amount of time it took for the participant to move his or her right foot from the accelerator to the brake pedal after the red light lit up on the driving simulator and did not distinguish between reaction time and brake time as in the study by Holt et al. [3, 11].

Our study is the first to investigate the influence of surgical shoes on BRT following hallux valgus deformity correction. The main strengths of our study are the relatively large number of patients, the standardized method, the follow-up over 8 weeks (2 weeks after full mobilization), and the fact that each of the 42 patients performed 20 BRT measurements per session (except for two patients at the 2-week measurement), giving a total of 5800 BRT measurements.

We acknowledge the following limitations of the present study, which may also be viewed as recommendations for future studies: foremost, we tested our patients under standardized conditions and it should be mentioned that driving safety is also influenced by external factors. Second, we tested only the effect of surgical shoes after right-sided first metatarsal osteotomy and did not investigate the left foot while driving with a left-foot adapter. Third, surgical shoes might not be prescribed by all orthopedic surgeons for 6 weeks after the operation.

## Conclusions

On the basis of our findings it is concluded that driving abstinence is recommended for 6 weeks after a right-sided first metatarsal osteotomy. However, patients should have sufficient recovery, exercise, and training before resuming driving a car, because safety is always a priority.

## Abbreviations

AOFAS: American Orthopaedic Foot and Ankle Society
BRT: brake response time
FRS: forefoot relief shoe
HVS: hallux valgus shoe
m: meter
msec: millisecond
SD: standard deviation
